# Predicting pulmonary hemorrhage in very low birth weight infants

**DOI:** 10.1038/s41390-025-04736-0

**Published:** 2026-01-24

**Authors:** Natasha Lalos, Sherry Kausch, Brynne Sullivan, Zachary Vesoulis

**Affiliations:** 1 Division of Newborn Medicine, Department of Pediatrics, Washington University School of Medicine, St. Louis, MO USA; 2 Division of Neonatology, Department of Pediatrics, University of Virginia School of Medicine, Charlottesville, VA USA

## Abstract

**Background:**

Pulmonary hemorrhage (PH) in very low birth weight (VLBW) infants is a catastrophic event with significant mortality. Predicting PH clinically is challenging, with no tools for early detection. Our objective was to identify candidate physiologic biomarkers using heart rate (HR) and pulse oximetry (SpO_2_) predictive of PH events in VLBW infants.

**Methods:**

We conducted a retrospective case-control study of VLBW infants from two level IV NICUs (2016–2023). We matched infants with PH to controls using gestational age, birth weight, and postnatal age. Taking HR and SpO_2_ data from 72 hours before and after PH diagnosis and age-matched times for controls, we calculated a pulse oximetry warning score (POWS) using hourly mean, skewness, kurtosis, and cross-correlation of HR and SpO_2_.

**Results:**

We analyzed 48 infants, 24 PH cases, and 24 controls, with similar characteristics. Infants who developed PH exhibited a three-fold rise in POWS starting 24 hours before PH diagnosis. Control infants showed no significant change in POWS.

**Conclusion:**

POWS, a predictive model for cardiorespiratory deterioration, showed a rise in predicted risk before the clinical diagnosis of PH. This suggests that cardiorespiratory deterioration signatures precede the clinical diagnosis of PH and can be recognized with a computer algorithm.

**Impact:**

- There are subtle vital sign changes that may indicate impending pulmonary hemorrhage in very low birth weight infants.
- Cardiorespiratory predictive monitoring could be useful for early warning of deterioration due to pulmonary hemorrhage.
- This study lays the foundation for the utility of predictive analytics for pulmonary hemorrhage prediction and suggests that a dedicated predictive model for pulmonary hemorrhage may be helpful.

## Introduction

Pulmonary hemorrhage (PH) is a catastrophic event with high morbidity and mortality in very low birth weight (VLBW) infants. Clinical presentation is defined by acute respiratory decompensation, frank red blood or frothy pink-tinged secretions in the endotracheal tube, and homogenous diffuse infiltrates on chest radiograph.^1^ Morbidities include bronchopulmonary dysplasia, increased length of mechanical ventilation, and increased length of hospital stay. Mortality from PH has been reported at up to 50% in published literature.^2–6^ The incidence of PH is 1 to 12 per 1,000 live births, with an increasing incidence (50 per 1000 live births) in high-risk populations such as the VLBW infants.^4–7^ Clinical factors that increase the likelihood of PH are commonly found in the neonatal intensive care unit (NICU) and are non-specific, including chorioamnionitis, intrauterine growth restriction (IUGR), respiratory distress syndrome (RDS), sepsis, disseminated intravascular coagulation (DIC), hypoxemia, surfactant replacement, intubation in the delivery room, and mechanical ventilation.^4,7,8^ Post-hemorrhage management practices include increased mean airway pressure (MAP), endotracheal tube epinephrine, intravascular volume replacement, and hemodynamic support. Despite these aggressive measures, studies have shown higher mortality for infants with PH at seven days (40.6% vs 18.9%), at 30 days (54% vs 28.8%), and at NICU discharge (56.9% vs 33.7%).^2–5^

We recently developed and validated a cardiorespiratory model to predict risk of deterioration due to sepsis and sepsis-like conditions in >3000 VLBW infants. The pulse oximetry warning system (POWS), uses features calculated from continuous heart rate (HR) and oxygenation (SpO_2_) data to capture changes in the distribution of these vital signs and their interaction.^9^ Given the acute respiratory deterioration seen in neonatal PH, we hypothesized that POWS or its component features may be useful for early warning of impending deterioration due to PH.

## Methods

### Setting

We performed a retrospective case-control study of vital sign data from VLBW infants admitted to two academic level IV NICUs: St. Louis Children’s Hospital (SLCH) and University of Virginia Children’s Hospital (UVA) between January 2016 and January 2023. The study was approved by the Institutional Review Boards at each site. Clinical and cardiorespiratory data from all VLBW admissions were collected at each site from the electronic medical record (EMR).

### Inclusion and exclusion criteria

PH was defined as clinical decompensation coupled with frank-red blood or blood-tinged secretions suctioned from the airway. Clinical decompensation included one or more of new-onset hypoxemia, hypercarbia, shock, and/or escalation of respiratory support from baseline as reported in the EMR. Infants were excluded if no vital sign data were stored, if they were admitted to the NICU beyond 3 days of age, or had major congenital anomalies.

### Control cohort matching

Following the selection of the PH cohort, we constructed a control cohort of VLBW infants without PH from the same two NICUs who met the same inclusion criteria, namely admission within 3 days of birth and vital sign data available. Control infants were matched to the PH group on gestational age within one week, birth weight within 100 g, and vital sign data available from a post-natal age matched to within 24 hours of the PH diagnosis time.

### Clinical data collection methods

We reviewed progress notes, problem list diagnoses, laboratory/imaging results, and flowsheets to record demographic and clinical data, including the mode of respiratory support and FiO_2_ charted prior to the onset of the event. We collected any relevant echocardiogram data available near the time of PH diagnosis, and the control matched time to compare rates of PDA diagnosis. In addition, we collected data on comorbidities that commonly occur during the same postnatal period as PH, including early or late-onset sepsis, intraventricular hemorrhage, necrotizing enterocolitis, and intestinal perforation. Sepsis outcomes were defined as culture-positive sepsis, clinical sepsis (defined as receiving >5 days of antibiotics in the setting of a negative blood culture), or sepsis ruled out. Furthermore, we evaluated the initial severity of illness using Clinical Risk Index for Babies (CRIB-II) scores^10^ and tracked early (7-day) and late (before NICU discharge) mortality.

To determine a definitive time of PH diagnosis (T_0_) we utilized the following multi-step approach: first, if a code sheet was available, the documented start time of the code event was used, if unavailable the documented medication administration time of endotracheal tube epinephrine was used, and if neither of the aforementioned were available, time of chest radiograph order was used as the best proxy for time of PH diagnosis.

### Vital sign collection methods

Vital sign data are continuously sampled every two seconds throughout NICU hospitalization and archived at both sites. HR and SpO_2_ data were obtained from the bedside monitors (NICU 1: Intellivue MP70 or MX800, Philips Medical, Andover, MA; NICU 2: CARESCAPE B850, GE Healthcare, Milwaukee, WI). Vital sign data were prospectively archived using BedMasterEX (Hillrom Medical Device Integration, Chicago, IL) from January 2016- December 2022 and T3 (Etiometry Inc, Boston, MA) from January 2023 to January 2024 at NICU 1. BedMasterEX (Hillrom, Chicago, IL) was used throughout the entire study during at NICU 2. The pulse oximetry device used at NICU 1 was the Nellcor OxiMax (Medtronic, Boulder, CO), and the Rad7 (Masimo, Irvine, CA) was used at NICU 2.

Using HR and SpO_2_ data from 72 hours before (T_-72_) and 72 hours after (T_+72_) the PH event time (T_0_), we calculated the mean, standard deviation, skewness, kurtosis of HR and SpO_2_ and their minimum and maximum cross-correlation in 10-minute windows. We then averaged the 10-min calculations hourly and used them to calculate POWS with methods previously described by Kausch, et al.^9^ While the model was originally trained to detect imminent late-onset sepsis, further studies have demonstrated the utility of POWS for early warning of cardiorespiratory deterioration with or without sepsis.^9,11^ POWS scores indicate the relative risk of deterioration from baseline. We calculated hourly POWS scores and evaluated its utility for PH detection by plotting POWS and the input features as a function of time to PH diagnosis.

### Typical clinical practices

At UVA, all infants born before 25 completed weeks of gestation are intubated in the delivery room. Infants at greater gestational ages are intubated based on the severity of respiratory distress. All infants intubated in the delivery room are given immediate prophylactic surfactant. Infants on non-invasive support but with FiO_2_ persistently > 0.5 have rescue surfactant administered via the LISA method. At SLCH, infants born before 25 completed weeks of gestation are also intubated in the delivery room, with more mature infants managed with non-invasive support as the primary approach. Infants at greater gestational ages are intubated in the delivery room for persistent FiO_2_ requirement exceeding 0.7 and/or inadequate spontaneous respiratory effort. Similar to UVA, all SLCH infants intubated in the delivery room are given immediate prophylactic surfactant. Infants admitted to the SLCH NICU on non-invasive support but with FiO2 ≥ 0.3 by 4 hours of life have rescue surfactant administered via the LISA method.

Other clinical practices were equally similar between the institutions. SpO_2_ alarm limits at UVA were 88-95% while they were 88–96% at SLCH. Both centers provide caffeine to infants born before 32 weeks of gestation with an initial bolus dose of 20 mg/kg followed by 10 mg/kg/day as a maintenance dose until 34 weeks corrected gestational age. At UVA, prophylactic indomethacin was given to all infants with a birth weight < 1000 *g* while at SLCH, prophylactic indomethacin was given to all infants born less than 27 completed weeks of gestation.

### Statistical methods

Cohort characteristics were summarized and compared for cases and controls using Student’s t-tests for continuous variables, Fisher’s exact tests for two-level categorical variables, and chi-square tests for multilevel categorical variables. POWS scores were compared between cases and controls at each of 12 discrete time points (T-72 to T + 72 hours) using Wilcoxon rank-sum tests. To account for multiple comparisons across timepoints we applied the Holm-Bonferroni sequential correction method, which controls the family-wise error rate at α = 0.05. We plotted POWS as a function of time to PH diagnosis (cases) or matched postnatal age (controls) and evaluated hourly POWS for a rise in score preceding clinical PH diagnosis. Finally, we calculated the area under the receiver operator characteristic curve (AUC) for POWS and individual POWS features to predict PH across multiple time horizons. All analyses were performed in R Studio version 2024.12.1 + 563 using the *ggplot2*^12^ and *pROC*^13^ packages.

## Results

### Cohort demographic and clinical characteristics

We identified 1358 VLBW infants admitted during the study period, of whom 53 were diagnosed with PH. We excluded 25 infants for missing data and 4 for major congenital anomalies. In total, 24 PH cases met all inclusion criteria and were successfully matched with 24 controls of similar demographic characteristics. Events occurred at a median postnatal age of 61 hours with an IQR of 39.8 to 122 hours. The mean gestational age and birth weight were not statistically different (26.0 vs 26.1 weeks; 749 vs 765 grams) between cases and controls, nor was their respiratory support or FiO_2_. CRIB-II scores were the same between cases and controls (12 vs. 12), but 7-day mortality was higher among PH cases than non-PH controls (29% vs. 4%, *p* = 0.02). Sepsis outcomes among both cohorts were similar. Among 18 cases and 17 controls (73% of the cohort total) with a postnatal echocardiogram, 13 cases (54%) and 9 controls (38%) were diagnosed with PDA. Most echocardiograms were performed after the time of PH diagnosis. A detailed overview of the clinical and demographic factors is found in Table 1.

**Table 1:** Patient and Clinical Characteristics

	Case (*n* = 24)	Control (*n* = 24)	*P* value
Gestational age (weeks), mean ± SD	26.0 ± 1.7	26.1 ± 1.6	0.83
Birth weight (grams), mean ± SD	749 ± 231	765 ± 226	0.73
Age at event (hours), median (IQR)	61 (39.8–122)	61 (39.8–122)	1.00
Female sex, *n* (%)	10 (42)	9 (38)	1.00
Race			
Black, *n* (%)	9 (38)	8 (33)	0.82
White, *n* (%)	14 (58)	14 (58)	
Unknown, *n* (%)	1 (4)	2 (8)	
Early onset sepsis outcomes^a^			
Culture positive sepsis	1 (4)	0 (0)	0.59
Clinical sepsis^c^	7 (29)	7 (29)	
Sepsis ruled out	16 (67)	17 (71)	
Late onset sepsis outcomes^b^			
Culture positive sepsis	1 (4)	0 (0)	0.32
Clinical sepsis^c^	7 (29)	4 (17)	
Sepsis ruled out	16 (67)	20 (83)	
Intraventricular hemorrhage (IVH), *n* (%)	9 (38)	6 (25)	0.53
Patent ductus arteriosus (PDA)			
Present, *n* (%)	13 (54)	9 (38)	0.36
Absent, *n* (%)	4 (17)	8 (33)	
Not assessed, *n* (%)	7 (29)	7 (29)	
Invasive support prior to event			
Yes, *n* (%)	19 (80)	15 (62)	0.66
No, *n* (%)	5 (20)	9 (38)	
Mode of respiratory support prior to event			
Bubble CPAP, *n* (%)	3 (13)	5 (21)	0.77
NIPPV, *n* (%)	2 (8)	4 (17)	
Conventional mechanical ventilation, *n* (%)	15 (63)	12 (50)	
High frequency jet ventilation, *n* (%)	2 (8)	1 (4)	
High frequency oscillatory ventilation, *n* (%)	2 (8)	2 (8)	
FiO_2_ prior to event, mean ± SD	0.51 ± 0.25	0.41 ± 0.23	0.14
Necrotizing enterocolitis (NEC), *n* (%)	2 (8)	3 (13)	1.00
Spontaneous intestinal perforation (SIP), *n* (%)	3 (13)	3 (13)	1.00
CRIB – II score, median (IQR)	12 (10-14)	12 (9.5-14)	0.94
Mortality within 7 days of event, *n* (%)	7 (29)	1 (4)	0.02*
Mortality before NICU discharge, *n* (%)	8 (33)	5 (21)	0.34

^a^Defined as sepsis before 72 hours of life, ^b^Defined as sepsis ≥72 hours of life, ^c^Defined as receiving > 5 days of antibiotics despite negative blood culture. *CRIB* Clinical Risk Index for Babies, *IQR* Interquartile Range (25th–75th percentile), *NIPPV* non-invasive positive pressure ventilation, *SD* standard deviation. Median values compared using Wilcoxon rank-sum test; categorical variables using Fisher’s Exact or Chi-square (>2 categories), mean values using t-test. *p < 0.05.

### POWS, HR, SpO_2_ changes in PH cases and controls

**Fig. 1:**
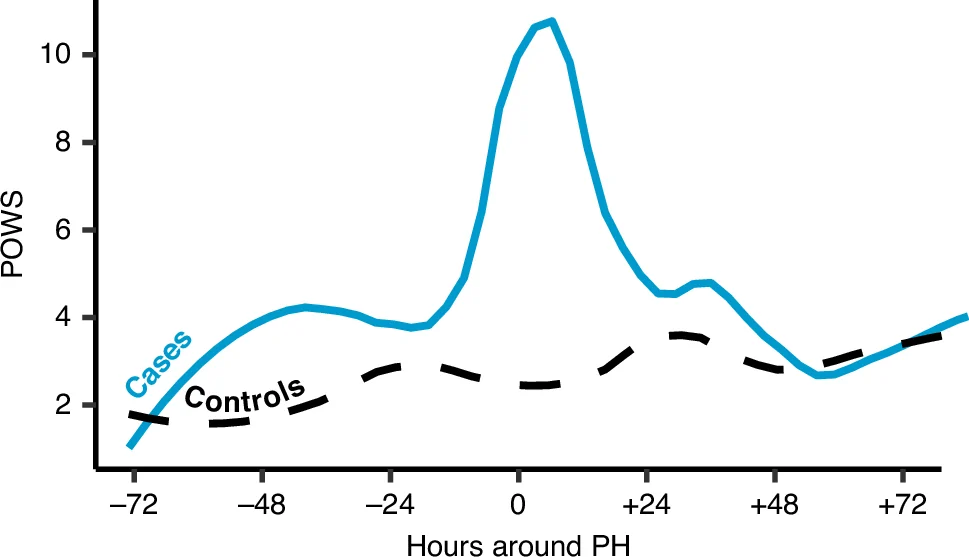
Cardiorespiratory deterioration score (POWS) over time from T-72 to T + 72. The light blue line represents cases, and the dashed black line represents controls.

Infants with PH had higher POWS scores at the start of the study window (T_-72_) than controls and demonstrated a continued rise in POWS predicted risk from T_-24_ to T_0_ while there was no change the postnatal age-matched period in controls (Fig. 1). Infants with PH had a dynamic rise in POWS from T_-24_ to T_0_ and fall from T_0_ to T_+24_, while there was no significant change over the same period in the control cohort (Fig. 1).

Median POWS was higher in cases than controls at all time points from T_-48_ until the event time T_0_. The median value of POWS at the time of PH diagnosis (T_0_) was 2.7-fold higher compared to 24 hours earlier (T_-24_ POWS = 2.02, T_0_ POWS = 5.37, *p* < 0.01) and was 4-fold higher compared to the median value of POWS at T_0_ for control (1.33). There was no significant change in median POWS during the study window (minimum score 1.22, maximum score 2.92) among controls from T_-72_ to T_+72._ A detailed summary of median and IQR of POWS in the PH and control groups is found in Supplementary Table S1.

**Fig. 2:**
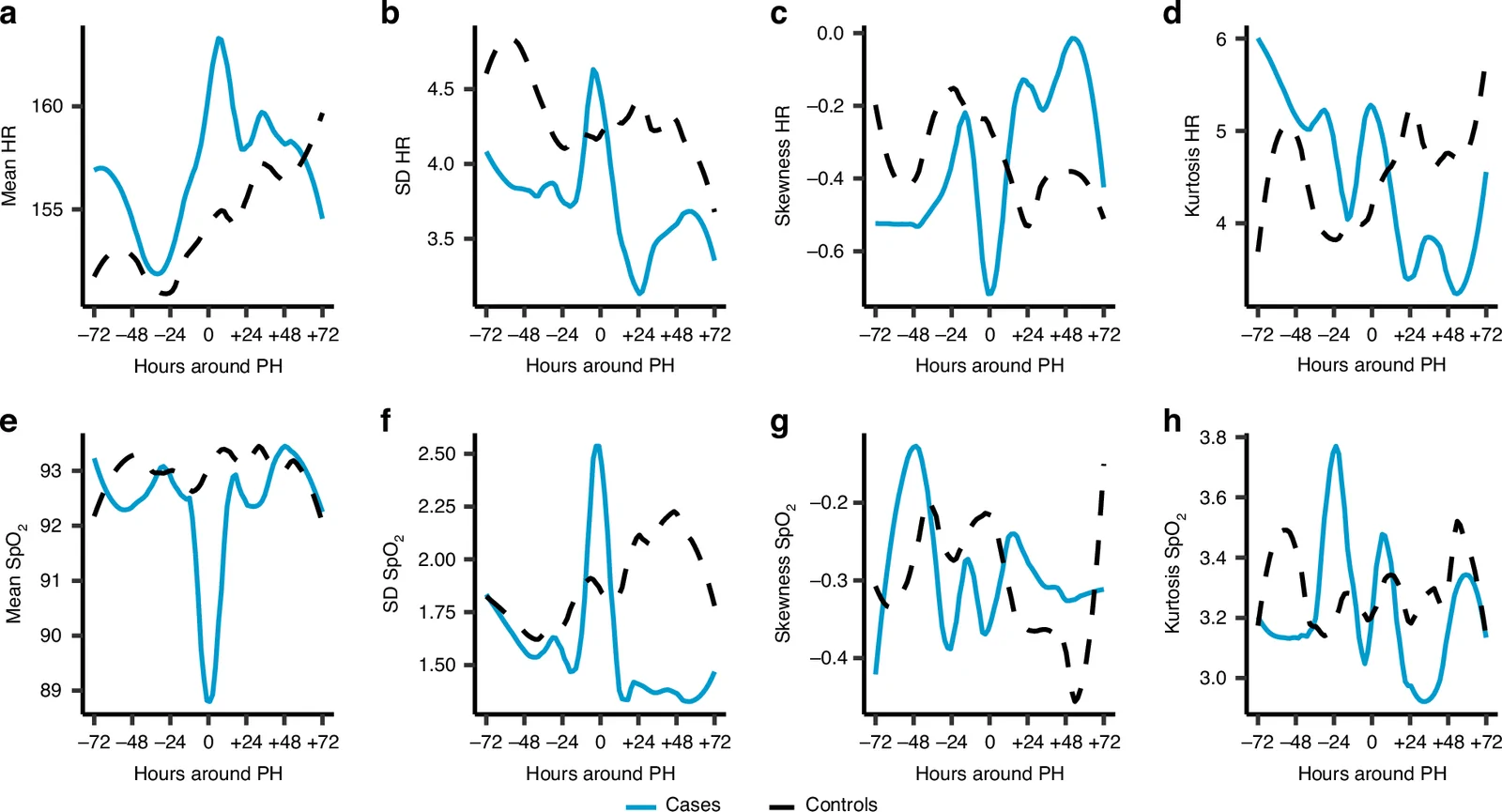
HR and SpO_2_ features calculated hourly and plotted as time to event for cases and age-matched time for controls. The top row shows HR characteristics, specifically **a** Mean HR, **b** SD HR **c** Skewness of HR, and **d** Kurtosis of HR. The bottom row shows SpO_2_ characteristics, including **e** Mean SpO_2_ **f** SD SpO_2_ **g** Skewness SpO_2_ and **h** Kurtosis SpO_2_. All plots show hours surrounding PH on the x-axis from −72 h to +72 hours. All plots show cases with the solid light blue line and controls with a dashed black line. Y-axis scales vary by plot.

When examining the individual HR and SpO_2_ features, mean HR increased, and skewness HR decreased near T_0_ for PH cases but fluctuated throughout the window for both cases and controls (Fig. 2a). Mean SpO_2_ decreased, and the standard deviation (SD) of SpO_2_ increased near T_0_ in PH cases but stayed relatively constant among controls (Fig. 2b).

### Discrimination of POWS and HR/SpO_2_ metrics by time to PH

**Fig. 3:**
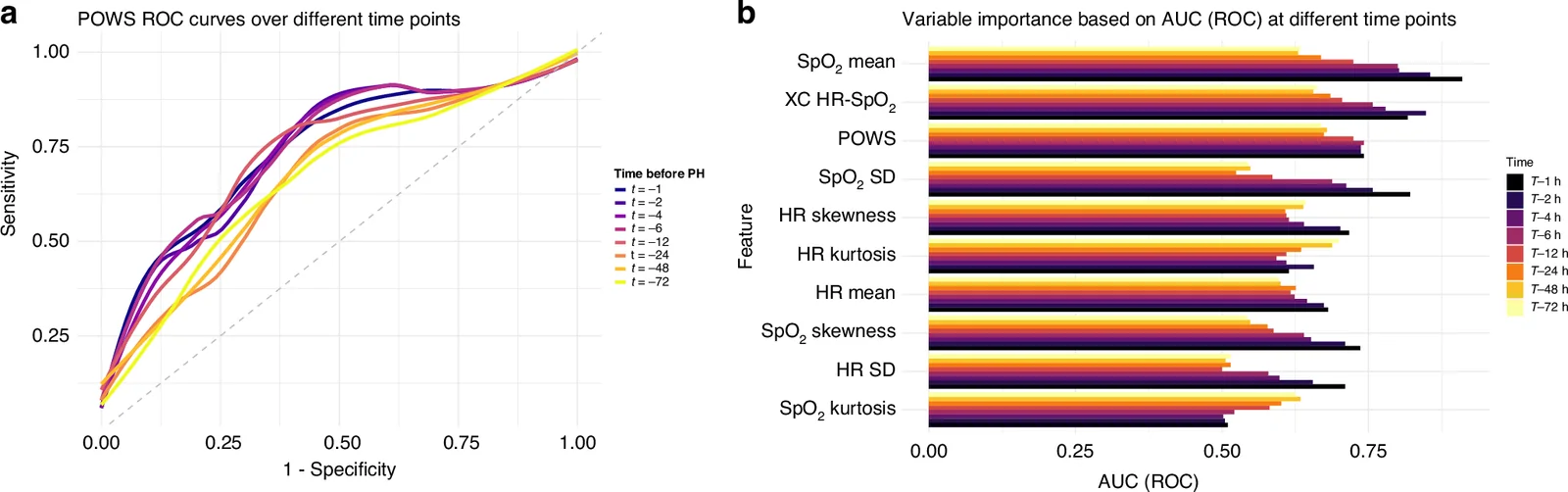
Discrimination of POWS and HR/SpO2 features of PH vs. controls by time to event. **a** shows receiver operator characteristic (ROC) curves for POWS to predict PH at eight timepoints relative to PH diagnosis and matched control times. **b** shows the area under the ROC curve (AUC) for HR and SpO_2_ features across the eight time points preceding PH diagnosis. Discrimination improves at timepoints closer to PH diagnosis.

Area under the receiver operator characteristic curve (AUC) was calculated for POWS to predict PH and  for HR and SpO_2_ features at the following time points: T_-72_, T_-48_, T_-24_, T_-12_, T_-6_, T_-4_, T_-2_ and T_-1_ (Fig. 3a and b, Table 2). Individual HR and SpO_2_ metrics demonstrated no (AUC 0.5-0.6) to poor discrimination (AUC 0.6–0.7) at T_-72_, T_-48_, and T_-24._ POWS and SD SpO_2_ had acceptable discrimination (AUC 0.7–0.8) at all time points between T_-12_ and T_-1_. Cross correlation of HR and SpO_2_ showed acceptable discrimination between times points T_-12_ to T_-4_ and excellent discrimination (AUC 0.8-0.9) between time points T_-2_ to T_-1_. Mean SpO_2_ demonstrated excellent discrimination at time points from T_-12_ to T_-1_. HR characteristics overall showed no to poor discrimination at all time points. Supplementary Fig. S2 shows AUC over time for each HR and SpO_2_ metric, illustrating mean SpO_2_ and HR-SpO_2_ cross-correlation as the best individual physiologic predictors of PH and better discrimination than POWS, which combines features.

**Table 2:** AUC measures for HR and SpO_2_ metrics over time

Feature	AUC T -1	AUC T -2	AUC T -4	AUC T -6	AUC T -12	AUC T -24	AUC T -48	AUC T -72
HR Characteristics								
Kurtosis	0.614	0.657	0.610	0.593	0.610	0.635	0.688	0.699
Mean	0.681	0.674	0.645	0.624	0.617	0.626	0.600	0.597
SD	**0.710**	0.655	0.598	0.579	0.500	0.515	0.506	0.515
Skewness	**0.717**	**0.702**	0.640	0.614	0.610	0.608	0.639	0.643
SpO_2_ Characteristics								
Kurtosis	0.510	0.505	0.502	0.521	0.581	0.601	0.634	0.626
Mean	**0.910**	**0.855**	**0.802**	**0.800**	**0.724**	0.669	0.630	0.634
SD	**0.821**	**0.757**	**0.712**	0.688	0.586	0.524	0.548	0.545
Skewness	**0.736**	**0.702**	0.652	0.640	0.588	0.578	0.548	0.543
Cross-correlation HR & SpO2	**0.817**	**0.848**	**0.779**	**0.757**	**0.705**	0.685	0.656	0.662
POWS	**0.742**	**0.737**	**0.737**	**0.742**	**0.724**	0.674	0.679	0.669

AUC table for characteristics across multiple time horizons preceding PH. Bold values indicate acceptable (or better) performance (AUC > 0.7).

## Discussion

In this study, we explored the potential use of physiologic predictive analytics to provide early warning of impending catastrophic events with subclinical preludes, such as PH in VLBW infants. Our primary focus was on the predictive value of POWS, an algorithm designed to detect cardiorespiratory signatures of illness. Our findings demonstrated a dynamic rise in POWS in the 24 hours preceding a PH diagnosis, indicating a potential window for earlier diagnosis and the possibility of early intervention.

The etiology of PH is multifactorial and heterogeneous. One theory hypothesizes that a contributing factor could be pulmonary over-circulation from a patent ductus arteriosus, leading to pulmonary congestion and increased transmural hydrostatic pressure on the fragile, easily broken pulmonary capillary bed.^14–16^ Still, there remains some debate as to the causative role of the PDA, as PH remains rare but PDA is common. Several studies have shown an association between PDA and PH, while others, including the Trial of Indomethacin Prophylaxis in Preterms (TIPP), failed to show any association.^17–20^ In our cohort, more infants with PH were also diagnosed with a PDA, but the retrospective nature of the study and small sample size did not enable us to draw any conclusions on the causative role of PDA in PH or the detected changes in POWS, HR, or SpO_2_. However, the poor performance of all HR-based characteristics in this model may indicate cardiac origins of PH are less likely, although more specific cardiac data are needed to make that conclusion.

Cardiorespiratory monitoring data contain patterns such as variability in beat-to-beat HR and SpO_2_, deceleration and acceleration in HR, and episodic instability in SpO_2._ These physiologic biomarkers reflect changes from normal physiology to the emergence of a pathophysiologic process. The quintessential demonstration of the power of predictive analytics was shown through the heart rate characteristic (HRC) monitor, now commercially available as the HeRO System (MPSC, Charlottesville, VA), for early warning of neonatal sepsis.^21–23^ Implementation of the HeRO monitor in a large, multicenter randomized clinical trial resulted in VLBW infant mortality reduction through earlier detection of critical illness.^24–29^ Predictive analytic models have also been developed in adult medicine to calculate the risk of impending respiratory failure and the need for emergent intubation through underlying physiologic vital sign instability along multiple time horizons preceding the event.^30–32^ This study represents the first application of this analytical approach to the prediction of pulmonary hemorrhage.

Our analysis revealed that SpO_2_ metrics outperformed the composite POWS index when predicting PH at all time points from T_-12_ to T_0_, whereas HR characteristics always underperformed. Given that SpO_2_ showed superior performance, it is plausible that we are detecting the pathophysiologic precursor to PH—pulmonary capillary congestion prior to hemorrhage with progressive impairment in oxygenation. Although the POWS algorithm was originally trained for the detection of sepsis, respiratory deterioration is part of the sepsis syndrome; thus, transfer learning to other forms of respiratory decompensation would likely be successful. Analysis demonstrating differential performance amongst elements suggests that an algorithm tailored specifically for PH diagnosis, placing greater weight on SpO_2_ characteristics and potentially incorporating other markers like mechanical ventilation (mean airway pressure, PEEP, FiO_2_), and/or PDA size, might be more effective than POWS or SpO_2_ metrics alone. Additionally, incorporating clinical variables and perinatal risk factors could further improve PH risk prediction. Large, multicenter cohorts would be necessary given the rare incidence of this condition.

We examined initial illness severity through the calculation of CRIB-II, a mortality risk index, for each patient. The CRIB-II score takes into account the birth weight, gestational age, body temperature, base excess, and sex of the baby within one hour of admission.^10^ CRIB-II scores were similar between cases and controls, suggesting no difference in illness severity or predicted mortality at the time of NICU admission. However, even though CRIB-II scores were similar for cases and controls, we identified that the baseline POWS scores were slightly higher in cases, suggesting that vital sign data may capture baseline physiologic inherent risk indicators not apparent in other metrics.

This study has several limitations, principally the small sample size and retrospective nature of the data. Even among VLBW infants, PH is a rare diagnosis, making it challenging to study despite a multi-year, multi-center effort. The retrospective nature of this study limited our ability to analyze contemporaneous clinical observations by bedside providers leading up to PH diagnosis. It is possible that subtle changes or events were observed but not recorded in the medical record, making the assigned time of PH diagnosis inaccurate. POWS is not a risk index specific to PH diagnosis but rather was initially developed for sepsis prediction. However, clinical illness in neonates is commonly manifested as cardiorespiratory instability, not specific to sepsis. It is increasingly apparent that POWS or physiology-based predictive monitoring could provide early warning of deterioration due to multiple conditions with overlapping signatures of illness in preterm infants. Due to the small cohort, inter-center variability in subjective vs. quantitative data in echocardiogram reports, and known concerns with interrater variability, we classified only the presence or absence of the PDA and not size or hemodynamic significance.

This study was strengthened by our early prediction of PH starting 24 hours prior to the event, robust available vital sign data, and a well-balanced case and control group. To the authors knowledge, this is the first evaluation of a dynamic physiology-based algorithm for the prediction of pulmonary hemorrhage in VLBW infants. All prior studies have focused on epidemiologic patterns and/or antenatal or clinical risk factors. Rather than the continuously updating risk score of POWS, these studies only provide static risk prediction or backward-looking predictions.

In conclusion, subtle changes in cardiorespiratory monitoring data occur in the period preceding clinical diagnosis of PH and can be detected by a computer algorithm. Predictive monitoring using physiology-based algorithms could prompt earlier assessment and intervention, offering the potential to alter the clinical course of this catastrophic condition.

## Supplementary information

Supplementary Materials PH paper v4.

## Data Availability

Vital sign data for this project were derived from a publicly available dataset hosted by the University of Virginia (https://doi.org/10.18130/V3/KMT8QR). Additional clinical metadata cannot be disclosed due to privacy rules.
