# Supplementary material for: Predicting pulmonary hemorrhage in very low birth weight infants
Source: Pediatr Res. 2026 Jan 24;100(2):751–6. doi: 10.1038/s41390-025-04736-0 (PMC12961956; doi:10.1038/s41390-025-04736-0)

Supplemental Table S1. POWS scores compared between cases and controls for mean, median and standard deviations

**Hours to event**	PH Cases		Controls		Adjusted P value
	Median	IQR	Median	IQR	
**T-72**	1.08	0.97-1.36	1.27	0.69-2.27	0.43
**T-48**	2.24	1.35-3.79	1.32	0.94-2.03	0.15
**T-24**	2.02	1.68-4.85	1.97	0.77-4.02	0.85
**T-12**	1.96	1.14-4.03	1.22	0.84-3.18	0.19
**T-6**	3.33	1.26-8.87	1.26	1.02-2.91	0.05
**T-4**	3.84	1.73-6.09	1.41	0.83-2.87	0.03*
**T-2**	3.97	2.09-8.49	1.78	0.93-3.40	0.04*
**T-1**	4.75	2.18-12.78	1.42	0.83-3.36	0.01*
**T0**	5.37	2.61-13.61	1.33	0.9-3.64	<0.01**
**T12**	1.89	1.23-6.17	1.33	0.71-3.28	0.22
**T24**	3.34	1.96-6.46	2.11	1.15-3.47	0.19
**T72**	6.55	2.15-7.82	2.92	1.58-5.68	0.39

*Footnote: POWS = Pulse Oximetry Warning Score; IQR = Interquartile Range (25th -75th percentile); PH = Pulmonary Hemorrhage. Time points represent hours relative to PH diagnosis (cases) or matched postnatal age (controls). Median values compared using Wilcoxon rank-sum test, p values adjusted for multiple comparison using the Holm-Bonferroni sequential correction method. *p<0.05, **p<0.01.*

Supplemental Figure S2. Variable importance based on AUC (ROC) at different timepoints

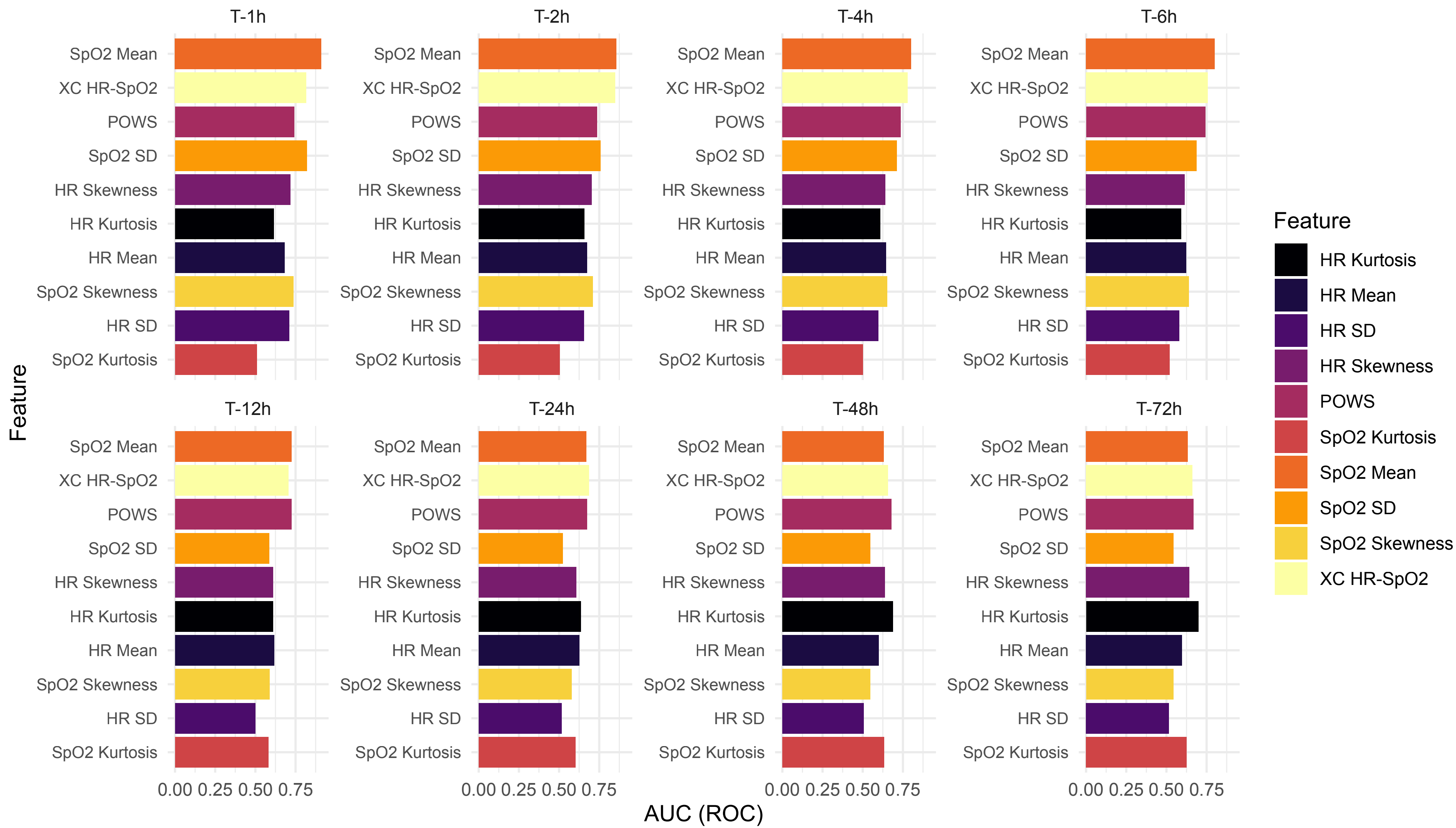

Supplement: Supplementary file 1 [file 41390_2025_4736_MOESM1_ESM.pdf]
